# A study of the clinical significance of mSEPT9 in monitoring recurrence and prognosis in patients with surgically treated colorectal cancer

**DOI:** 10.1371/journal.pone.0312676

**Published:** 2024-10-28

**Authors:** Rong Li, Jiaojiao Chen, Xin Shen, Yanping Lin, Jiadai Tang, Guangrui Xiong, Ke Zhang, Mengying Xiang, Lin Xie, Fengdi Hu

**Affiliations:** 1 Department of Digestive Neoplasms, Peking University Cancer Hospital Yunnan, The Third Affiliated Hospital of Kunming Medical University, Yunnan Cancer Hospital, Kunming, Yunnan Province, China; 2 Radiotherapy Department, The First Affiliated Hospital of Hainan Medical University, Haikou, Hainan Province, China; 3 Gastroenterology, Lincang People’s Hospital, Lincang, Yunnan Province, China; 4 Department of Oncology, Baoshan People’s Hospital in Yunnan Province, Baoshan, Yunnan Province, China; 5 Department of Critical Care Medicine, Peking University Cancer Hospital Yunnan, The Third Affiliated Hospital of Kunming Medical University, Yunnan Cancer Hospital, Kunming, Yunnan Province, China; University Magna Graecia of Catanzaro, ITALY

## Abstract

**Objective:**

To explore the medical significance of methylated septin9 (mSEPT9) in monitoring recurrence and prognostic assessment in individuals with surgically treated colorectal cancer (CRC).

**Methods:**

To investigate the role of Septin9 in colorectal cancer, we utilized the TIMER2.0 database to analyze its differential expression between tumor tissues and adjacent normal tissues. Colorectal cancer RNA-seq data from the TCGA database was downloaded and curated. The clinical relevance of mSEPT9 in colorectal cancer was explored by examining the correlation between Septin9 methylation levels and clinical characteristics using UALCAN and MethSurv software. Peripheral blood samples were obtained from 130 CRC subjects who underwent surgery for the detection of mSEPT9 and carcinoembryonic antigen (CEA) expression, along with collection of clinicopathological data such as age, gender, tumor site, TNM stage, and tumor differentiation. Patients were followed up for at least 3 years post-surgery until the death or final follow-up dates (31/12/2022). Additionally, peripheral blood samples were collected from 30 colorectal cancer surgery patients for mSEPT9 detection before and 7 days after surgery.

**Results:**

Through bioinformatic database analysis, we identified higher expression levels of SEPT9 mRNA in most tumor tissues compared to normal tissues. Similarly, both paired and unpaired CRC tissues exhibited elevated expression of Septin9 when compared to normal tissues. Following GO and KEGG analysis of Septin9 target genes, we discovered their significant associations with ncRNA metabolic processes, ribonucleoprotein complex biogenesis, spliceosomes, and viral carcinogenesis. Furthermore, the overexpression of mSeptin9 was observed in CRC tissues, and it demonstrated a correlation with colon cancer staging and histologic classification. In our clinical sample study, The positive rate of mSEPT9 in CRC patients 7 days after surgery was 43.44% lower than that of preoperative. The differences in TNM stage, tumor differentiation degree, and preoperative CEA expression level between the preoperative mSEPT9 positive and negative groups of CRC were statistically significant (*P* < 0.05). Recurrence free survival (RFS) and overall survival (OS) were shorter in the preoperative mSEPT9-positive group, meaning preoperative mSEPT9 status was a risk factor for CRC recurrence and prognosis (*P* < 0.05). The sensitivity, specificity, and AUC value of preoperative mSEPT9 and CEA levels for predicting postoperative recurrence in CRC patients were 88% vs. 72%, 56.19% vs. 55.24%, and 0.721 vs. 0.636 respectively, well the AUC value of the combined prediction of postoperative recurrence was 0.758.

**Conclusion:**

The detection of mSEPT9 combined with CEA in preoperative plasma helps predict recurrence in colorectal cancer patients.

## Introduction

Colorectal cancer (CRC) ranked as the third most prevalent form of malignancy globally and stood as the second primary contributor to cancer-related mortality [1]. In China, it is noteworthy that CRC ranks as the fourth most significant contributor to mortality, specifically in relation to deaths related to cancer. It is worth mentioning that both the occurrence and death rates of CRC have shown a consistent upward trajectory over the years [2]. About 20% of newly diagnosed individuals with CRC had distant metastases, with a rate of 5-year survival that is fewer than 20% [3]. Currently, the therapeutic drugs and the treatment strategies for CRC are continuously developing and optimized. However, 30% to 50% of CRC patients still experience recurrence or metastasis after surgery, which is the most important reason for death [4]. Right now, there is still a lack of better methods for evaluating the effects of surgical treatment and predicting the risks of recurrence of CRC. Even though carcinoembryonic antigen (CEA) and CT examination are the main testing methods adopted in clinical practice for postoperative regular follow-up monitoring, the sensitivity and specificity of CEA are not high enough. They could only be used as a certain degree of reference in clinical practice. On the other hand, CT could not be repeated frequently due to radiation and expensive price, and some tiny recurrent lesions were still undetectable. With the advancement of second-generation sequencing technologies, it has been found that the detection of microscopic residual lesions (MRDs) plays an important role in postoperative monitoring and guiding treatment. Until now, the method of the detection technology applied to the clinic has not yet been completely unified in various studies due to its high price, which is not suitable for large-scale investment in the clinic for real-time monitoring of the patient’s condition changes [5,6]. Therefore, it’s truly necessary to continue exploring a safe, reproducible, highly sensitive, and specific detection technique for observing the recurrence of postoperative CRC.

DNA methylation was a vital epigenetic modification, and CpG islands were the major locations of DNA methylation. DNA methylation led to transcriptional silencing and inactivation of oncogenes, which was an important process in early carcinogenesis. Studies have identified that DNA methylation is closely connected with the progression of various malignant tumors such as CRC, hepatocellular carcinoma, breast malignancy, etc. [7]. The SEPT9 gene is situated on chromosome 17q25.3 and has broad expression in human cells. The results of methylation assay studies at tissue and plasma levels showed that the SEPT9-v2 promoter region was almost completely methylated in CRC and precancerous patients [8]. It has been informed that the sensitivity and specificity of methylated septin9 (mSEPT9) for identifying CRC could be as high as 76.6% and 95.9%, respectively [9,10]. Plasma detection of mSEPT9 for screening CRC has already been successfully utilized at home and abroad [11]. However, there were still fewer studies on the importance of mSEPT9 in CRC postoperative recurrence monitoring and prognostic determination.

In this investigation, We initially conducted an analysis of the expression of the Septin9 gene in cancer utilizing bioinformatics methods, investigated the potential functions of Septin9 target genes, and examined the correlation between the methylation level of Septin9 and the clinicopathological characteristics of CRC using bioinformatics databases. Then, we evaluated mSeptin9 expression in clinical samples of CRC collected from our center. Our aim was to analyze the correlation between mSEPT9 and clinicopathological characteristics of patients, as well as investigate the impact of mSEPT9 on progression-free survival (PFS) and overall survival (OS) in individuals with CRC. Preliminarily analyzing the clinical value of mSEPT9 on the CRC recurrence and prognosis could provide a theoretical basis for exploring the research of monitoring the clinical indexes for the determination of recurrence and prognosis of CRC patients.

## Materials and methods

### Bioinformatic analysis

We analyzed the differential expression of Septin9 in tumor tissues and adjacent normal tissues using TIMER2.0 database (http://timer.cistrome.org/). We also obtained RNAseq data from TCGA database (382 CRC tissues) and GTEX database (358 normal tissues) for unpaired sample comparisons. Additionally, we downloaded RNAseq data from TCGA-COAD and TCGA-READ (50 paired cases) and collated them with the previous data in FPKM format for further analysis of Septin9 expression in CRC. We explored the clinical significance of Septin9 in CRC by investigating the relationship between Septin9 methylation levels and clinical features of CRC using UALCAN and MethSurv in TCGA samples.

### Subjects and study design

**Fig 1:**
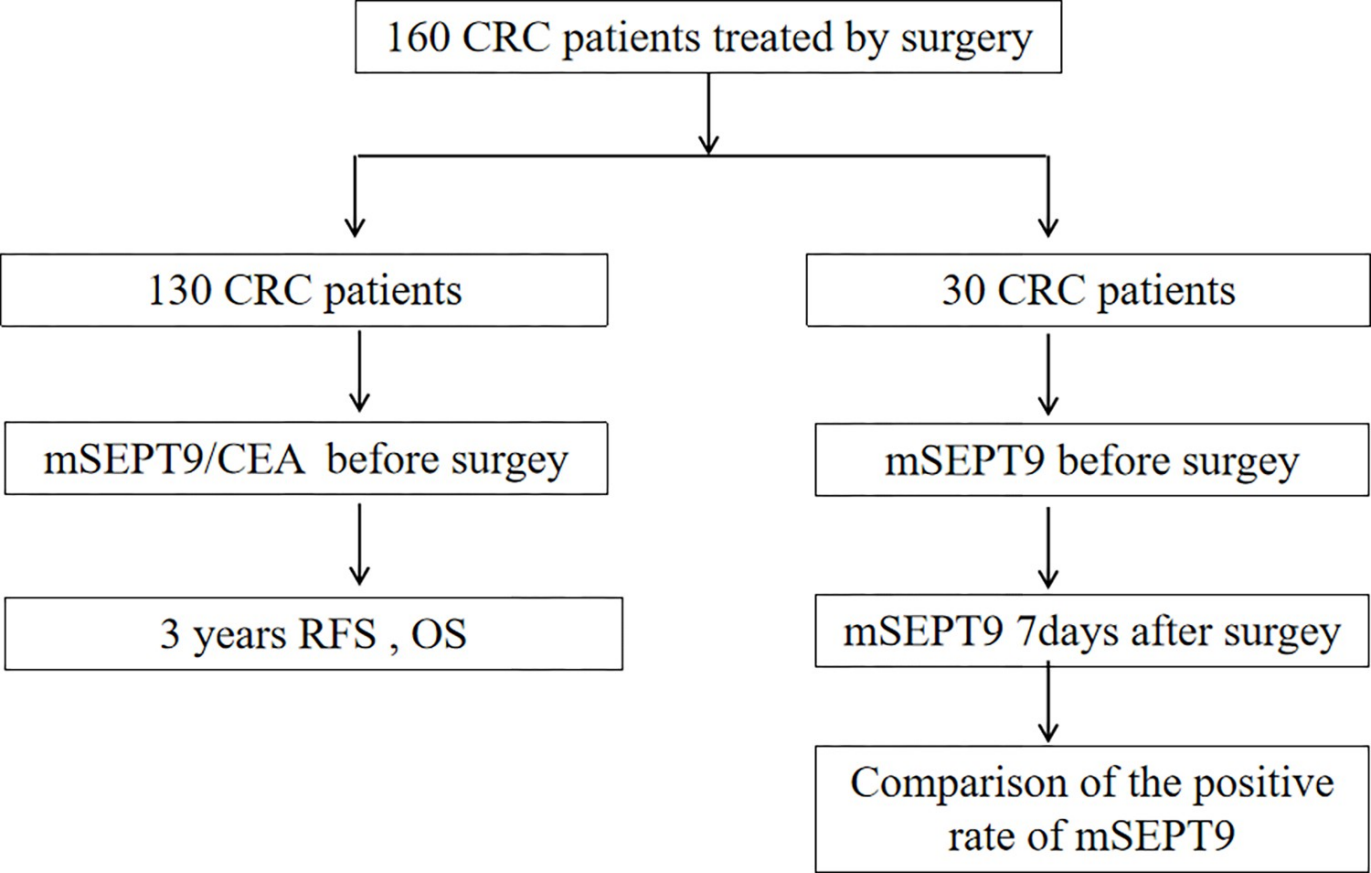
Sample collection flowchart.

We collected 160 CRC patients who underwent initial surgical treatment without any preoperative antitumor therapy between January 2018 and December 2018 at Tumor Hospital of Yunnan Province. They included stage I-III and initially resectable stage IV patients with at least 3 years of postoperative follow-up until the death or final follow-up dates (31/12/2022). Recurrence in this study included local recurrence and distant metastasis. The investigation was authorized by the Research Ethics Committee of the Third Affiliated Hospital of Kunming Medical University, and written informed consent was acquired from all participants (kylx2022082). The specific process is shown below (Fig 1).

### Sample collection and storage

In 130 CRC patients, 10ml of peripheral venous fasting blood was obtained in the early morning before surgery, and another 30 patients were recruited to acquire 10 ml of fasting peripheral venous blood twice in the early morning before and 1 week after surgery, respectively. The serum was placed in anticoagulated tubes containing K2EDTA (BD biosciences, NJ, USA), centrifuged to extract the serum, and stored in a -20°C refrigerator.

### mSEPT9 methylation quantification

In this study, a kit of SEPT9 methylation identification (BioChain, Beijing, China) was employed. Firstly, DNA was isolated from plasma utilizing a plasma extraction kit, then unmethylated cytosine was converted into uracil by deamidation reaction through incubation of the DNA with bisulfite, followed by PCR using a PCR kit with specific primers to make the sulfite-converted DNA (Bis-DNA) PCR amplification using a PCR kit with specific primers to amplify the sulfite-converted DNA (Bis-DNA), followed by detection of the target fragment in the SEPT9 gene using a fluorescent probe that specifically binds to it. SEPT9 methylation in plasma samples was detected by ABI 7500 fluorescent PCR using β-Actin gene as internal reference, and positive findings were defined as having a Ct value of ≤41.0, whereas negative outcomes were characterized by a Ct value of >41.0 or the inability to identify a Ct value.

### CEA quantification

The results were determined by radioimmuneofluorescence assay. CEA>5ng/mL was determined as positive.

### Statistical analyses

Statistical analysis was conducted employing SPSS 26.0, and statistical plots were drawn by GraphPad Prism 9.0. The correlation between mSEPT9 and clinicopathological features of participants was examined using a chi-square test, and survival analysis was dependent on recurrence free survival (RFS) and OS. RFS was determined as the time from surgery to local recurrence or distant metastasis in individuals with CRC. OS was known as the time from diagnosis of CRC to the end of CRC-related death or follow-up. The effects of single factors on RFS and OS were analyzed employing the Kaplan-Meier technique. COX multifactorial regression was utilized to analyze independent risk factors affecting patients’ prognosis.

## Results

### 1. Bioinformatics analysis of Septin9 and mSeptin9

**Fig 2:**
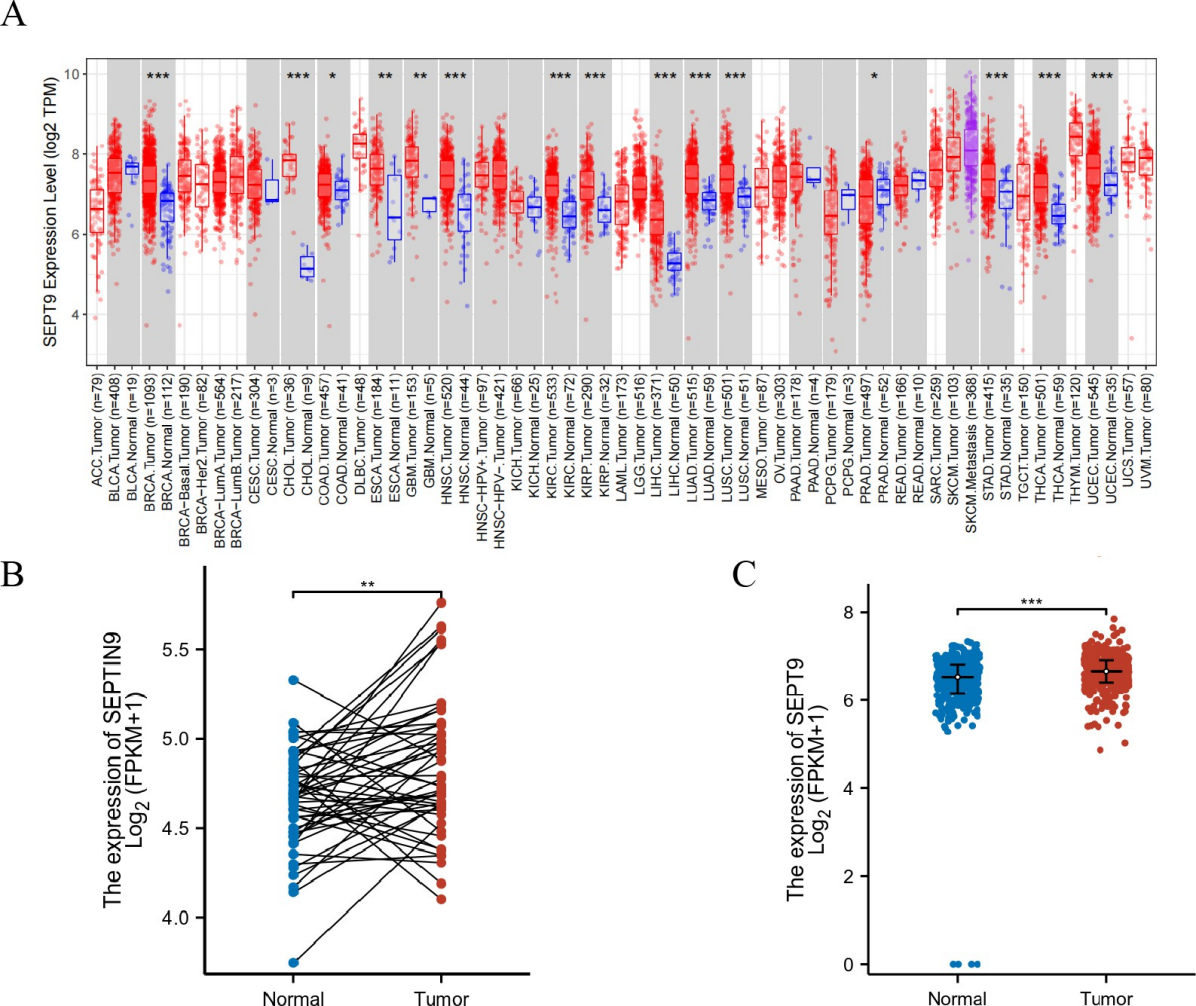
Expression of Septin9 in various cancer tissues, including CRC. A. Expression profiles of Septin9 in various types of cancer tissues. B. Expression profiles of Septin9 in paired CRC tissues. C. Expression profiles of Septin9 in unpaired CRC tissues. (*P<0.05, **P<0.01, ***P<0.001).

We conducted differential expression analysis of tumor tissues and adjacent normal tissues using the TIMER2.0 database. Our findings showed that the expression level of Septin9 mRNA was higher in tumor tissues compared to normal tissues in various cancer types, such as COAD, BRCA, CHOL, ESCA, HNSC, KIRC, LUAD, LUSC, PRAD, STAD, THCA, and UCEC (Fig 2A, *P*<0.05). In order to further investigate the expression of Septin9 in CRC tissues, we obtained RNAseq data of 382 CRC tissues from the TCGA database and 358 normal tissues from the GTEX database for unpaired sample analysis using XENA. Additionally, we collected RNAseq data of 50 paired cases from the TCGA database in TCGA-COAD and TCGA-READ. Our analysis revealed that the expression of Septin9 was higher in both paired and unpaired CRC tissues compared to normal tissues (Fig 2B and 2C, *P*<0.01).

**Fig 3:**
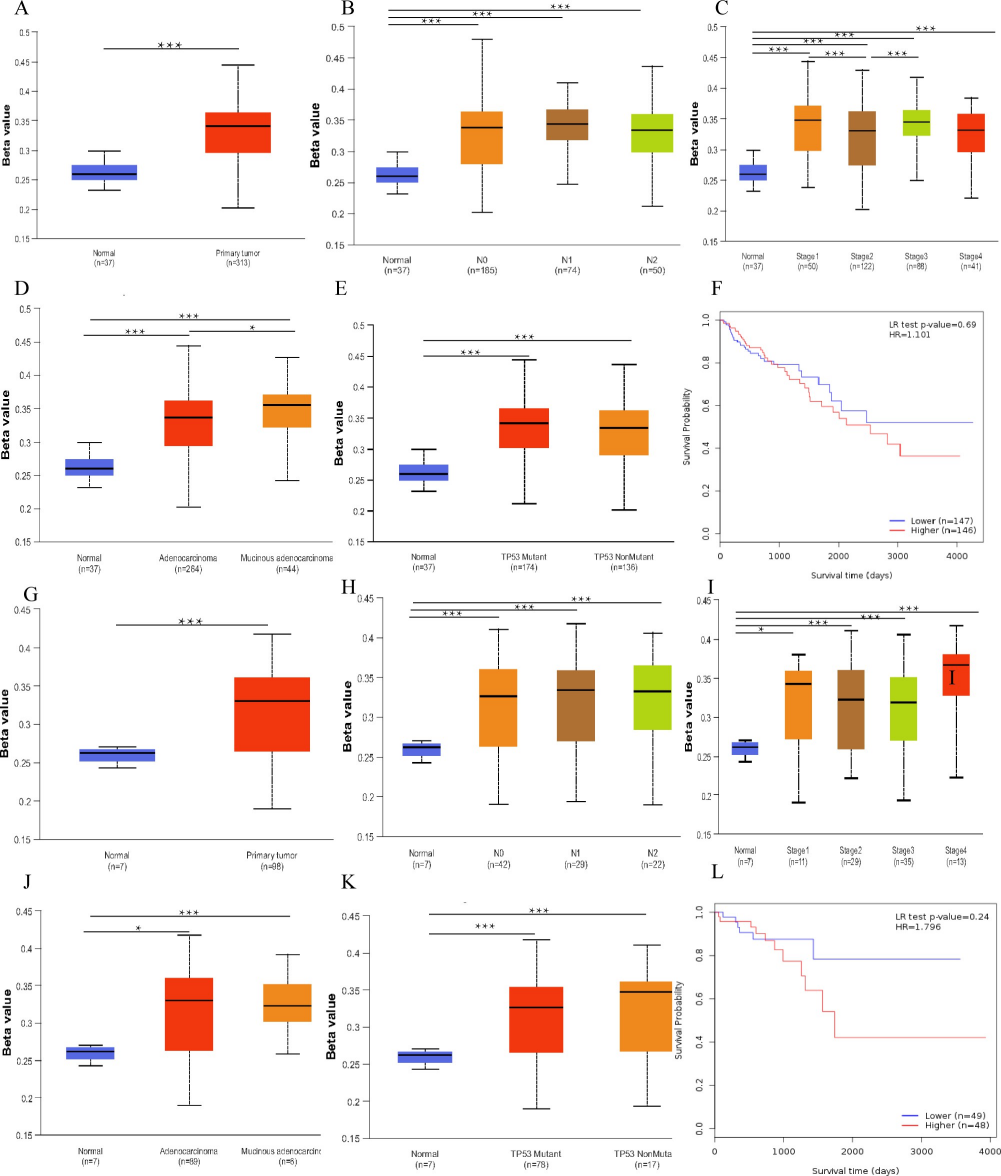
Relationship between mSeptin9 expression and clinical features of CRC and its Kaplan-Meier plot. A. mSeptin9 expression in colon cancer. B. mSeptin9 expression in colon cancer based on lymph node infiltration. C. mSeptin9 expression in colon cancer based on clinical stage. D. mSeptin9 expression in colon cancer based on histologic subtypes. E. mSeptin9 expression in colon cancer based on TP53 mutation status. F. Kaplan-Meier plot of mSeptin9 levels versus OS in patients with colon cancer. G. mSeptin9 expression in rectal cancer. H. mSeptin9 expression based on lymph node infiltration in rectal cancer. I. mSeptin9 expression based on clinical stage in rectal cancer. J. mSeptin9 expression based on histologic subtype in rectal cancer. K. mSeptin9 expression based on histologic subtype in rectal cancer. L. Kaplan-Meier plot of mSeptin9 levels versus OS in patients with rectal cancer. (*P<0.05, **P<0.01, ***P<0.001).

In order to further investigate the clinical significance of mSeptin9 in CRC, we conducted an analysis of TCGA samples using UALCAN and MethSurv to explore the relationship between the methylation level of Septin9 and the clinical characteristics of CRC. The results show significant hypermethylation of Septin9 in both colon and rectal cancers (Fig 3A and 3G, *P* < 0.001). Additionally, we examined mSeptin9 expression in relation to lymph node infiltration, cancer stage, histologic type, and TP53 mutation status (Fig 3B–3E and 3H–3K). Our findings revealed higher mSeptin9 expression in advanced-stage colon cancer patients (Fig 3C, *P<*0.001) and increased expression in mucinous adenocarcinoma compared to adenocarcinoma tissues (Fig 3D, *P<*0.05). Furthermore, we conducted survival analysis based on the methylation level of Septin9 categorized into high and low groups, and found no significant difference in survival rates between the mSeptin9 high-expression group and the low-expression group in CRC patients (Fig 3F and 3L, *P* > 0.05). However, it should be noted that the data was only collected up to 2017, which may present certain limitations.

### 2. Correlation of mSEPT9 with clinicopathological characteristics of CRC

The connection between clinicopathological features and mSEPT9 in 130 CRC patients, age, gender, and tumor location of CRC patients did not vary significantly in the preoperative mSEPT9-negative and positive groups (*P* > 0.05), and the differences between TNM staging, the level of differentiation, and the level of preoperative CEA expression were statistically significant in the two groups (*P* < 0.05) (Table 1).

**Table 1:** Correlation between clinicopathologic characteristics and mSEPT9 in 130 cases of CRC.

		mSEPT9 status			
	No. of patients	Negative	Positive	χ^2^	*P*
**Gender**					
Male	81	36	45	0.909	0.34
Female	49	26	23		
**Age**					
≤60 years	71	34	37	0.002	0.961
>60 years	59	28	31		
**BMI** (**kg/㎡)**					
<24	93	45	48	0.063	0.801
≥24	37	17	20		
**Localization**					
Colon	50	21	29	1.055	0.304
Rectum	80	41	39		
**Tumor differentiation**					
Poorly differentiated	34	9	25	8.312	0.004
Well and moderately differentiated	96	53	43		
**TNM stage**					
I/II	51	33	18	9.738	0.002
III/IV	79	29	50		
**CEA (ng/ml)**					
≤5	65	41	24	12.334	*<*0.001
>5	65	21	44		

### 3. Changes in plasma mSEPT9 in patients with CRC before and 7 days following operation

**Fig 4:**
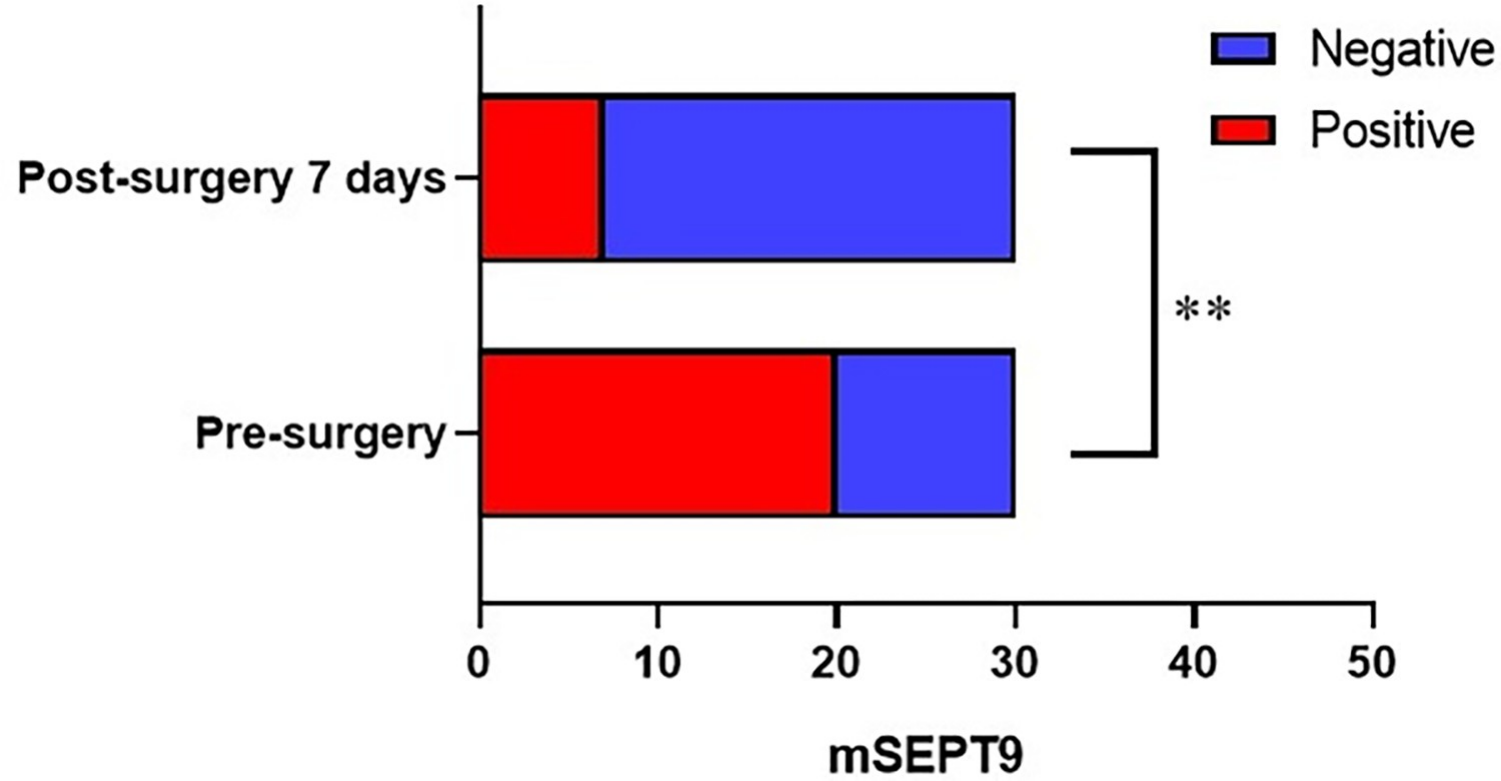
Changes in plasma mSEPT9 in individuals with CRC prior to and 7 days after operation. (**P<0.01).

We performed mSEPT9 testing on 30 patients 1 week prior to surgery and 7 days following surgery. Of the 20 individuals with CRC having positive plasma mSEPT9 prior to surgery, 13 turned negative 1 week after surgery, 7 were persistently positive, and 10 were persistently negative, and plasma mSEPT9 positivity rate in the week after surgery was significantly decreased compared to that of the preoperative period (23.33% vs. 66.67%), with a statistically significant variation (*P*<0.05, Fig 4).

### 4. Cox regression for RFS in CRC patients

Among CRC patients treated with stage I-IV surgery, univariate analysis showed that preoperative mSEPT9 positivity (HR: 9.297, 95% CI: 2.77–31.199, *P*<0.05), CEA>5 ng/ml (HR: 3.021, 95% CI: 1.26–7.242, *P*<0.05), late TNM staging (HR: 19.251, 95% CI: 2.6–142.522, *P* < 0.05) were connected with shorter RFS in patients. Good tumor differentiation (HR: 0.358, 95% CI: 0.162–0.789, *P* < 0.05) was associated with longer RFS, and there was no significant connection between sex, age, BMI, primary cancer site, and RFS in CRC patients (*P* > 0.05). Multifactorial analysis showed that mSEPT9 positivity (HR: 5.143, 95% CI: 1.425–18.559, *P*<0.05), TNM stage (HR: 11.49, 95% CI: 1.515–87.136, *P*<0.05) were predictive independent factors for RFS in subjects with CRC. mSEPT9 positivity with CEA>5 ng/ml was also a predictive independent risk factor (HR: 2.788, 95% CI: 1.167–6.662) (Table 2).

**Table 2:** Unifactorial and multifactorial analysis of RFS in CRC patients.

Variable	Recurrence-free survival (RFS)			
	Univariate		Multivariate	
	RFS HR (95% CI)	*P*	RFS HR (95% CI)	*P*
**Gender**				
Male	1 (ref)	0.714		
Female	0.859 (0.379–1.944)			
**Age**				
≤60 years	1 (ref)	0.236		
>60 years	0.61 (0.269–1.382)			
**BMI (kg/㎡)**				
*<*24	1 (ref)	0.681		
≥24	1.193 (0.515–2.764)			
**Localization**				
Colon	1 (ref)	0.221		
Rectum	1.726 (0.721–4.135)			
Tumor differentiation				
Poorly differentiated	1 (ref)	0.011	1 (ref)	0.308
Well and moderately differentiated	0.358 (0.162–0.789)		0.657 (0.294–1.472)	
**TNM stage**				
I/II	1 (ref)	0.004	1 (ref)	0.018
III/IV	19.251 (2.6–142.522)		11.49 (1.515–87.136)	
**mSEPT9 status**				
Negative	1 (ref)	*<*0.001	1 (ref)	0.012
Positive	9.297 (2.77–31.199)		5.143 (1.425–18.559)	
**CEA (ng/ml)**				
≤5	1 (ref)		1 (ref)	
≤5+mSEPT9 positive	3.035 (0.672–13.706)	0.149		
>5	3.021 (1.26–7.242)	0.013	1.442 (0.571–3.643)	0.439
>5+ mSEPT9 positive	4.306 (1.851–10.018)	0.001	2.788 (1.167–6.662)	0.021

**Fig 5:**
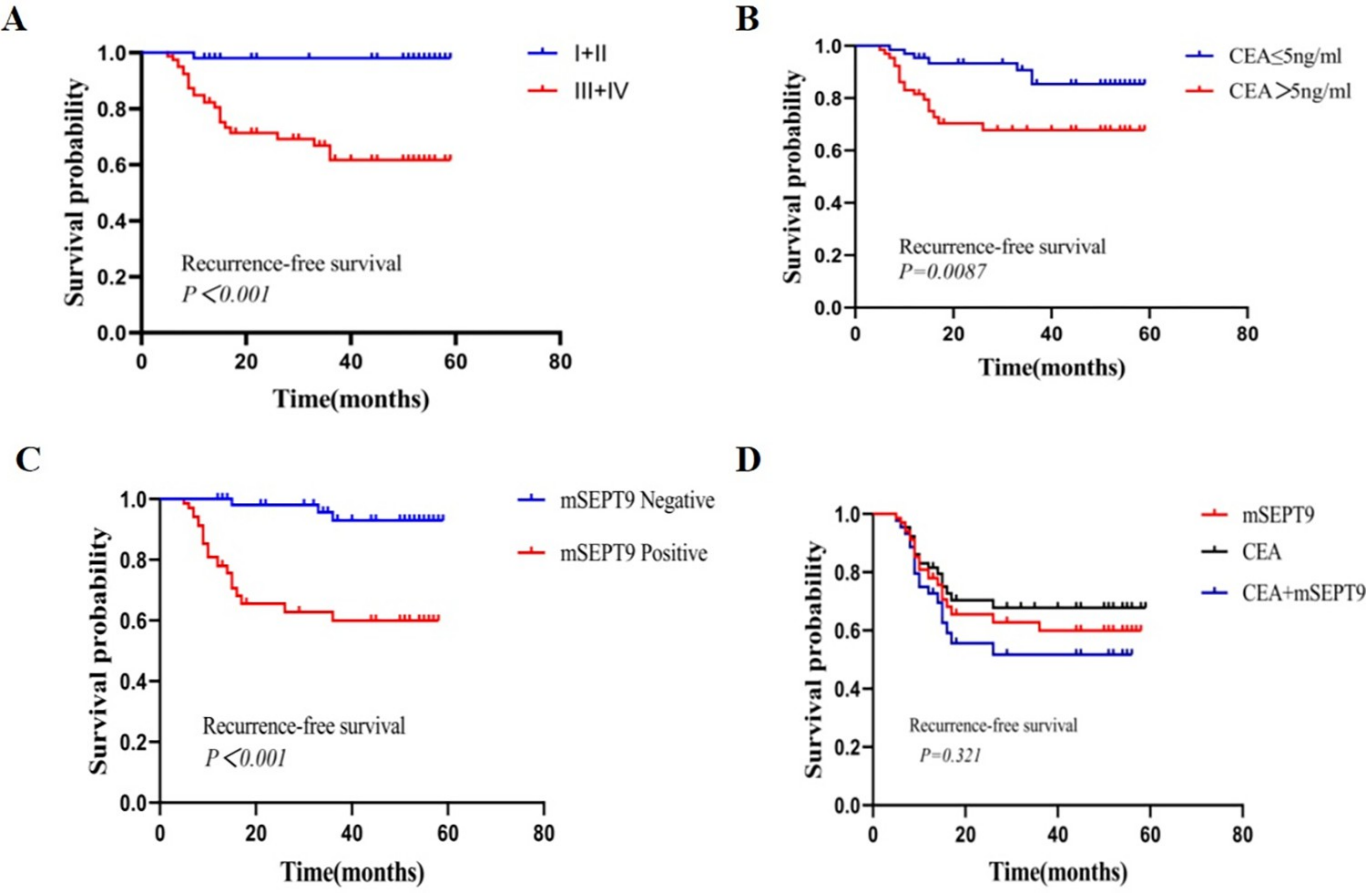
Kaplan-Meier curves for RFS depending on TNM status, mSEPT9, and CEA expression in CRC patients. A. Kaplan-Meier curves for RFS in accordance with TNM status. B. Kaplan-Meier curves for RFS depending on CEA expression. C. Kaplan-Meier curves for RFS depending on mSEPT9. D. Kaplan-Meier curves for RFS depending on mSEPT9 combined with CEA expression.(*P<0.05, **P<0.01, ***P<0.001).

Furthermore, Kaplan-Meier survival analysis suggested that the RFS of cases with preoperative CEA greater than 5 ng/ml and mSEPT9 positivity was significantly shorter than that of cases with CEA ≤5 ng/ml and mSEPT9-negativity, respectively (*P* < 0.01). In patients positive for both carcinoembryonic antigen (CEA) and methylated septin 9 (mSEPT9), there was a tendency towards shorter recurrence-free survival (RFS) compared to patients positive for CEA alone or mSEPT9 alone. However, the observed difference between the groups did not reach statistical significance (*P*>0.05) (Fig 5).

### 5. Cox regression for OS in CRC patients

In individuals with surgically treated CRC, univariate analysis suggested that compared with patients with preoperative mSEPT9-negative, preoperative mSEPT9-positive (HR: 5.41, 95% CI: 1.773–16.505, *P* < 0.05), CEA > 5 ng/ml (HR: 4.611, 95% CI: 1.516–14.027, *P* < 0.05), and TNM staging (HR: 20.091, 95% CI: 2.654–152.112, *P* < 0.05) were connected with shorter OS. There was no significant relationship between sex, age, BMI, primary cancer site, degree of differentiation, and OS in CRC patients (*P* > 0.05). Multifactorial analysis suggested that patients with late TNM staging (HR: 14.957, 95% CI: 1.849–120.956, *P* < 0.05), CEA > 5 ng/ml, and mSEPT9-positive patients (HR: 5.321, 95% CI: 1.702–16.636, *P* < 0.05) had a worse prognosis, which was an independent predictive risk factor for CRC patients (Table 3).

**Table 3:** Unifactorial and multifactorial analysis of OS in CRC patients.

	Overall survival (OS)			
	Univariate		Multivariate	
	OS HR (95% CI)	*P*	OS HR (95% CI)	*P*
**Gender**				
Male	1 (ref)	0.985		
Female	0.991 (0.384–2.559)			
**Age**				
≤60 years	1 (ref)	0.725		
>60 years	1.182 (0.465–3.003)			
**BMI (kg/㎡)**				
*<*24	1 (ref)	0.518		
≥24	0.693 (0.228–2.107)			
**Localization**				
Colon	1 (ref)	0.346		
Rectum	0.638 (0.251–1.624)			
**Tumor differentiation**				
Poorly differentiated	1 (ref)	0.718		
Well and moderately differentiated	1.257 (0.363–4.35)			
**TNM stage**				
I/II	1 (ref)	0.004	1 (ref)	0.011
III/IV	20.091 (2.654–152.112)		14.957 (1.849–120.956)	
**mSEPT9 status**				
Negative	1 (ref)	0.003	1 (ref)	0.366
Positive	5.41 (1.773–16.505)		1.798 (0.504–6.412)	
**CEA (ng/ml)**				
≤5	1 (ref)		1 (ref)	
≤5+mSEPT9 positive	0.756 (0.078–7.307)	0.809		
>5	4.611 (1.516–14.027)	0.007	3.371 (0.99–11.48)	0.052
>5+ mSEPT positive	8.118 (2.632–25.043)	*<*0.001	5.321 (1.702–16.636)	0.004

**Fig 6:**
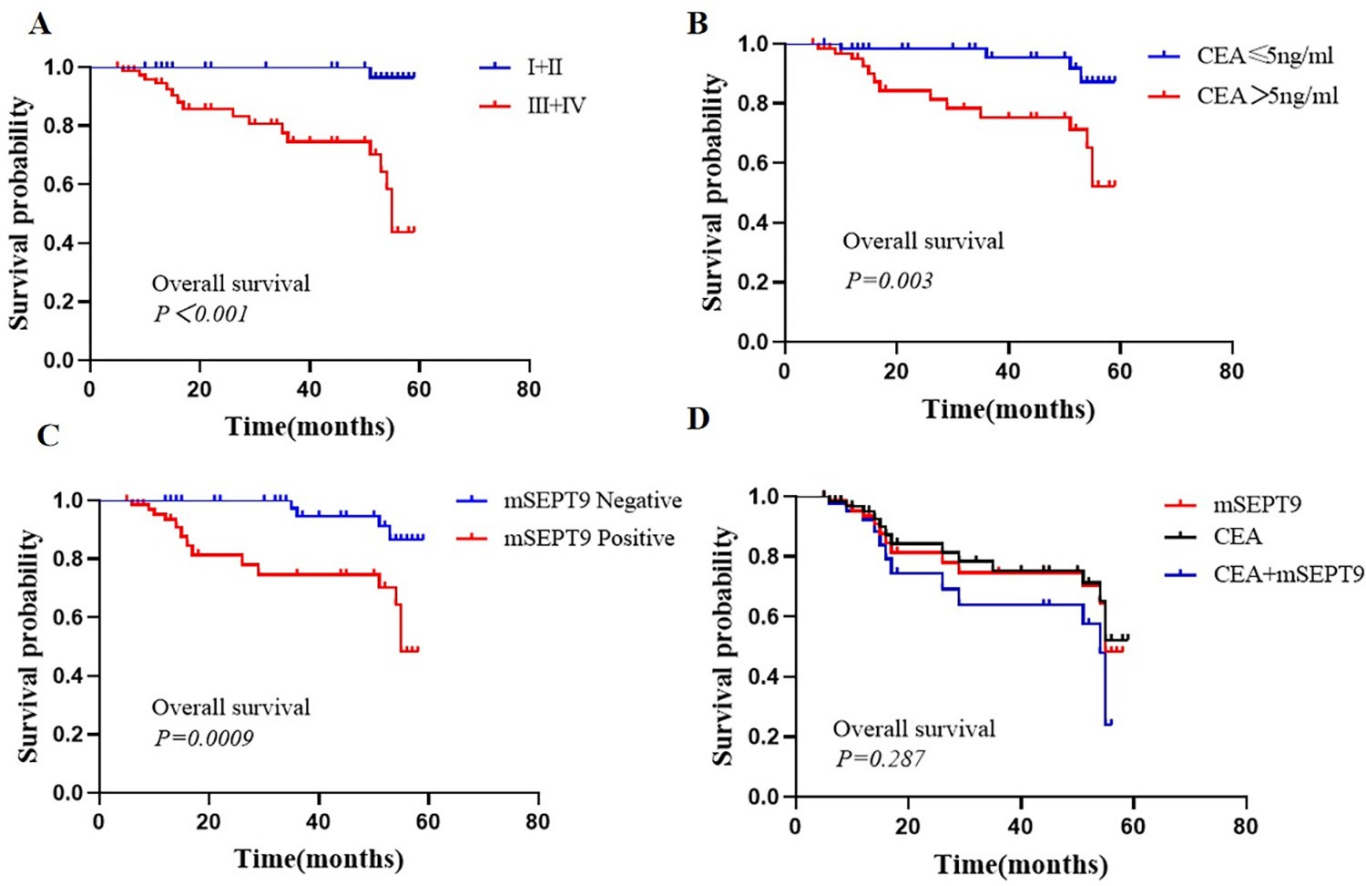
Kaplan-Meier curves for OS depending on TNM status, mSEPT9, and CEA expression in subjects with CRC. A. Kaplan-Meier curves for OS depending on TNM status. B. Kaplan-Meier curves for OS according to CEA expression. C. Kaplan-Meier curves for OS in accordance with mSEPT9. D. Kaplan-Meier curves for OS based on mSEPT9 combined with CEA expression. (*P<0.05, **P<0.01, ***P<0.001).

Furthermore, Kaplan-Meier survival analysis suggested that OS was significantly shorter in preoperative CEA >5 ng/ml, mSEPT9-positive patients than in CEA ≤5 ng/ml, mSEPT9-negative patients, respectively (*P* < 0.01). Patients positive for both CEA and mSEPT9 exhibited a trend towards shorter OS compared to those positive for CEA alone or mSEPT9 alone, although the observed difference was not statistically significant (*P* >0.05) (Fig 6).

### 6. Analysis of the predictive value of preoperative mSEPT9 and serum CEA levels on postoperative recurrence in individuals with CRC

**Fig 7:**
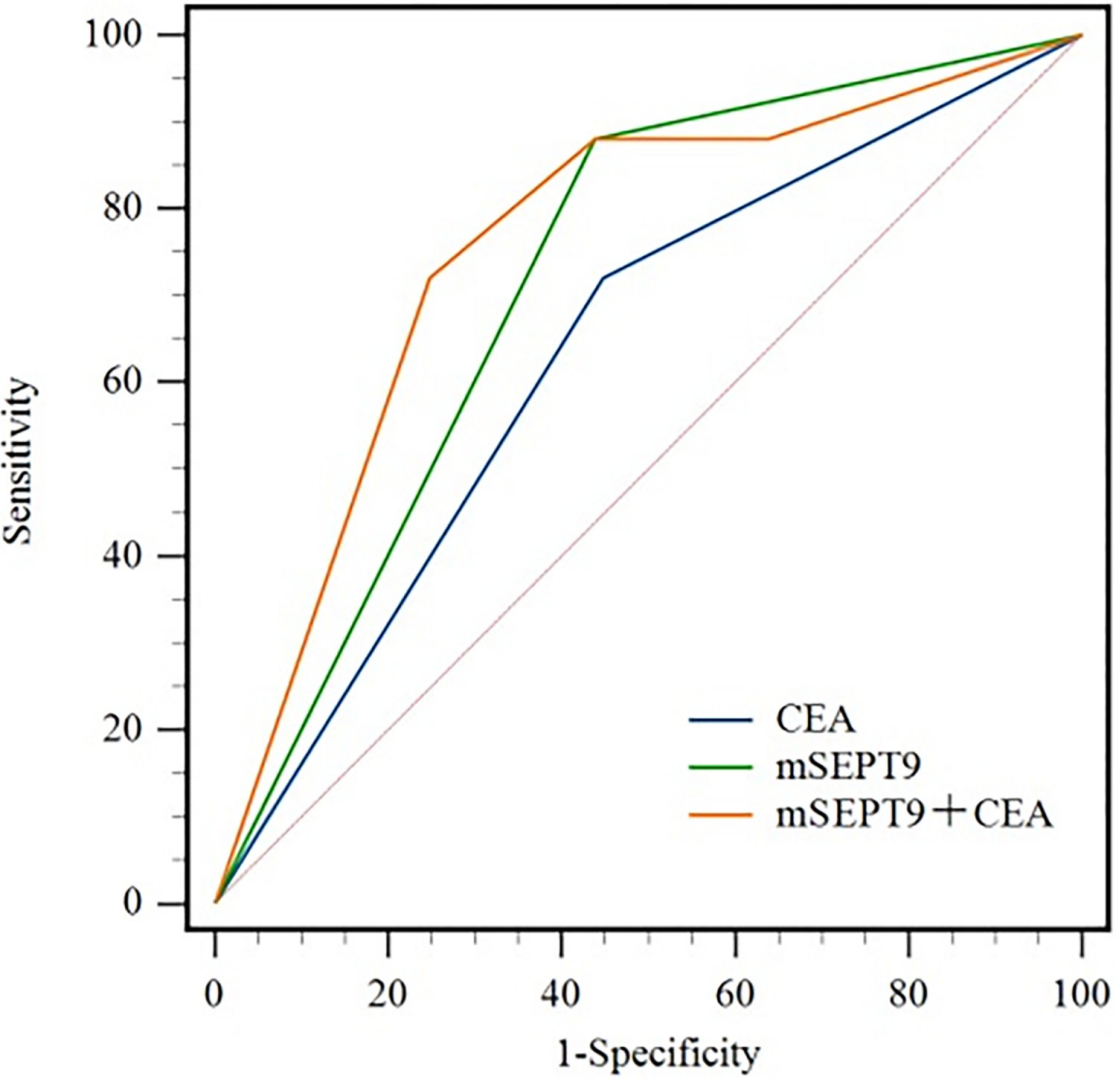
ROC curves of preoperative mSEPT9 and serum CEA levels for monitoring postoperative recurrence in CRC patients.

Prior to the operation, the sensitivity of mSEPT9 and CEA levels for the anticipation of postoperative recurrence in individuals with CRC was 88% and 72%, respectively, and the specificity was 56.19% and 55.24%, with area under the curve (AUC) values of 0.721 and 0.636, respectively. The AUC value of the combined prediction of postoperative recurrence by preoperative mSEPT9 and CEA was 0.758, with sensitivity and specificity of 72% and 75.24%, respectively. 75.24% and the AUC value and specificity were higher than those of each index alone (Fig 7).

## Discussion

At present, several studies have confirmed the remarkable mSEPT9 sensitivity and specificity in the diagnosis of CRC, and it is one of the most important indicators for clinical screening of colorectal adenoma(s) and carcinoma(s) [12–14]. However, the clinical value of mSEPT9 in CRC for monitoring postoperative recurrence and prognostic judgment has not been fully elucidated.

Recently, it has been shown that the methylation SEPT9 level is positively connected with tumor size and clinical stage of CRC but not with gender or age. Nearly 90% of preoperative mSEPT9-positive patients turned negative after surgery, and 10% of the patients who did not turn negative for mSEPT9 after surgery showed recurrence and metastasis sooner during the follow-up period [15]. A total of 51 individuals diagnosed with stage IV CRC and liver metastases were enlisted for a study. The researchers conducted measurements of mSEPT9 and CEA blood levels in these patients both before the surgical procedure and seven days post-surgery. The findings revealed that mSEPT9 and CEA were identified before having surgery in 92.2% (47/51) and 70.6% (36/51) of the subjects, respectively. Following the implementation of both simultaneous and phased surgical procedures, there was a significant decline in mSEPT9 levels by 923-fold and 11-fold, respectively. CEA level has shown a substantial decrease of 17-fold and 1.7-fold after both simultaneous and staged operations, respectively. Furthermore, the results of the survival study indicated that patients who tested negative for mSEPT9 both before and after undergoing the operation exhibited a more favorable survival rate compared to those who tested positive [16]. This finding suggests that mSEPT9 has the potential to function as a prediction marker. We examined the correlation between Septin9 methylation levels and clinical characteristics of CRC using UALCAN and MethSurv in TCGA samples. Our analysis revealed that mSeptin9 exhibited increased expression in CRC compared to normal tissues, and it was associated with the stage and histological classification of the disease. In our clnical study, we also found that the mSEPT9 positivity rate in CRC patients was 43.44% lower at 7 days postoperatively compared with preoperatively, which suggests that mSEPT9 has a certain clinical application in evaluating the response to surgical treatment of CRC.

A study recruited 83 patients with radically resected CRC and 16 patients with postoperative recurrence of CRC to undergo mSEPT9, fecal occult blood test (FOBT), CEA, and carbohydrate antigen 19–9 (CA19-9) testing, respectively. The outcomes indicated that the mSEPT9 sensitivity in diagnosing recurrence was much better than CEA, FOBT, and CA19-9, and the integration of mSEPT9 and CEA revealed an AUC of 0.978 for CRC recurrence [17]. It has also been informed that the pre-treatment mSEPT9 level in subjects with CRC is not only correlated with the TNM staging and Dukes’ staging but also associated with the MMR. The highest detection rate of mSEPT9 positivity in Dukes stage B may be related to the fact that deficient mismatch repair (dMMR) occurs predominantly in Dukes stage B, suggesting that dMMR may enhance SEPT9 hypermethylation. The study also recruited 70 patients with radically resected CRC to assess the medical value of mSEPT9, CEA, CA19-9, CA724, and contrast-enhanced computed tomography (CECT) in monitoring recurrence. mSEPT9 sensitivity and specificity to be 71.4% and 98.0%, respectively, which was greater than cancer biomarkers but lesser than CECT, which exhibited that single mSEPT9 identification or the integration of mSEPT9 and CECT were useful for observing the CRC recurrence [18]. In addition, some studies discovered the connection between mSEPT9 and medical features, mismatch repair proteins, and mutation status of CRC. The results showed that mSEPT9 was connected with dMMR, TNM stage, and mutations in BRAF, TP53, and PIK3CA in CRC. mSEPT9 with a Ct value of less than or equal to 37.5 was significantly correlated with poorer cancer-specific survival (CSS). mSEPT9 may influence the correlation of dMMR BRAF and PIK3CA mutations with CSS in CRC. In stage I-III postoperative bowel cancer, the positive mSEPT9 rate was linked to a shorter time to recurrence (TTR), and the sensitivity was greater than CEA. The prognostic value of mSEPT9 in combination with CEA was superior to mSEPT9 alone [19]. Our study indicates statistically significant differences in TNM stage, tumor differentiation, and preoperative CEA levels between the mSEPT9-positive and negative groups for colorectal cancer (CRC). Patients who tested positive for preoperative mSEPT9 exhibited shorter recurrence-free survival (RFS) and overall survival (OS). Those who were positive for both preoperative CEA and mSEPT9 had a worse prognosis compared to those positive for either marker alone. The combination of CEA and mSEPT9 significantly enhances the specificity and area under the curve (AUC) for recurrence prediction, providing moderate diagnostic value. Thus, mSEPT9 testing, either alone or combined with CEA, may serve as a useful marker for monitoring recurrence and assessing prognosis post-CRC surgery. However, our bioinformatics analysis of TCGA data did not reveal a significant correlation between mSEPT9 expression and survival outcomes, contrasting sharply with our clinical findings. In our cohort, preoperative mSEPT9 positivity was notably associated with shorter RFS compared to cases with CEA ≤5 ng/ml and mSEPT9 negativity. This discrepancy highlights the need for validating bioinformatics predictions in clinical settings. Differences in patient demographics, treatment approaches, tumor heterogeneity, and the time gap between the TCGA data and our study may account for this variance. A thorough discussion of these factors and the limitations of both methods is crucial to elucidate the clinical significance of mSEPT9 in CRC. Some studies have also focused on the medical importance of mSEPT9 in the monitoring of treatment side effects. For example, SEPT9 has been reported to be associated with vincristine-induced neurotoxicity [20]. The clinical application value of mSEPT9 in CRC still needs to be further explored. For example, the guiding significance of the combined detection of mSEPT9 and gene mutation status in the selection of treatment regimens and the clinical value of efficacy prediction in CRC patients.

There are plenty of studies about the clinical applicability value of mSEPT9 in malignant tumors, and some scholars have also conducted mechanism exploration studies for the role of SEPT9. Upon analysis of the bioinformatic database, we observed higher expression levels of SEPT9 mRNA in most tumor tissues compared to normal tissues. Additionally, both paired and unpaired CRC tissues exhibited elevated expression of Septin9 compared to normal tissues. At present, it is generally believed that SEPT9 gene is mainly related to the incidence and progression of CRC through 2 pathways. As the 4th cytoskeleton component, the lack of expression of SEPT9 protein will lead to the failure of cells to carry out cytoplasmic division to form multinuclei, and the cellular gene is unstable, which will lead to the occurrence of cancerous changes in the tissues [21], in addition, as an oncogene, high methylation of the CpG site will cause its gene expression to be blocked, and the oncogenic function will be lost. In addition, as an oncogene, the high degree of methylation at the CpG locus will block its gene expression and cause the impairment of its oncogenic role [22]. Studies have reported that SEPT9 promotes breast cancer EMT through activation of the FAK/Src signaling pathway by negatively regulating the ARHGAP4 protein in the Rho-GAP family [23]. In vitro and in vivo experiments have shown that SEPT9 and SEPT2 are able to activate both p53/p21 and MEK/ERK pathways to enhance the growth and invasion of glioblastoma cells [24]. Studies have shown that overexpression of SEPT9 attenuates myofibroblast biomarkers α-SMA and Col1a1 expression induced by TGF-β1, goes together with the upregulation of proteins associated with apoptosis, and slows down activation of hepatic stellate cells, so drugs that suppress the methylation and elevate the expression of SEPT9 hold promise for clinical application in the management of hepatic fibrosis [25]. The specific pathway of SEPT9 in CRC remains to be further deeply explored.

## Conclusion

Our study suggests that peripheral blood mSEPT9 can be utilized as an effective biomarker for monitoring recurrence and evaluating prognosis after surgery for CRC and that, combined with CEA testing, can improve the mSEPT9 diagnostic efficacy in the surveillance of recurrence in CRC. However, our study has restrictions such as a small sample size and a single-center trial. In the next study, we will carry on to enlarge the sample size for a multicenter trial and collect patients’ gene statuses of MSI, RAS, BRAF, PIK3CA, and Her2 to analyze the relationship between mSEPT9 and common mutated genes of bowel cancer, and to establish a diagnostic model for the surveillance of bowel cancer postoperative recurrence, with the aim of providing more bases for the selection of individualized postoperative treatment. In addition, we also plan to explore the medical importance of mSEPT9 in predicting the drug therapy effect in unresectable bowel cancer in order to find more effective biomarkers for clinical prediction of efficacy.

## Supporting information

S1 File (XLSX)
